# Distinct Patterns of Dyadic Mental Health in Patients with End-Stage Liver Disease and Their Care Partners

**DOI:** 10.3390/healthcare14050645

**Published:** 2026-03-04

**Authors:** Lissi Hansen, Karen S. Lyons, Nathan F. Dieckmann, Michael F. Chang, Shirin O. Hiatt, Susan J. Rosenkranz, Christopher S. Lee

**Affiliations:** 1 School of Nursing, Oregon Health and Science University, Portland, OR 97239, USA; dieckman@ohsu.edu (N.F.D.); hiatts@ohsu.edu (S.O.H.); rosenkra@ohsu.edu (S.J.R.); 2 William F. Connell School of Nursing, Boston College, Chestnut Hill, MA 02467, USA; lyonskw@bc.edu (K.S.L.); leeddo@bc.edu (C.S.L.); 3 VA Portland Health Care System, Portland, OR 97239, USA; michael.chang2@va.gov; 4 School of Medicine, Oregon Health and Science University, Portland, OR 97239, USA

**Keywords:** liver diseases, trajectory, caregivers, mental health, social support, palliative care, holistic approach

## Abstract

**Highlights:**

**What are the main findings?**

- Mental health is interdependent in end-stage liver disease and affects both members of the care dyad.
- Three distinct and different patterns of dyadic mental health were found over 12 months.

**What are the implications of the main findings?**

- Care partners who experience worse mental health than patients with end-stage liver disease should be the focus in dyadic intervention studies and clinical care.
- Early referral to palliative care is necessary.

**Abstract:**

**Background/Objectives**: Little research has examined changes over time in mental health within end-stage liver disease (ESLD) patient–care partner dyads. Therefore, the aim of this observational study was to identify patterns of dyadic mental health over time in a sample of ESLD dyads and associations with individual- and dyadic-level characteristics. **Methods**: Adult men and women with ESLD and their care partners were recruited at liver clinics at two healthcare centers in the U.S. Pacific Northwest. Survey data were collected at the time of study enrollment and at 3, 6, 9, and 12 months. Patients and care partners completed the Mishel Uncertainty in Illness Scale, the Multidimensional Perceived Social Support Scale, the Mutuality Scale, the Short-Form Health Survey, and one religiosity item. Standard summary statistics and multilevel and latent growth mixture modeling were used to analyze the data. **Results**: In total, 186 dyads were included in the analyses, which revealed three distinct patterns of dyadic mental health: “disparate: patient better” (*n* = 47 [25.3%]), “shared mental health” (*n* = 76 [40.86%]), and “disparate: care partner better” (*n* = 63 [33.87%]). Significant characteristics associated with the patterns included care-related strain, uncertainty, relationship quality, and social support. **Conclusions**: Clinical implications include greater attention to both members of the dyad, with particular attention to low levels of mental health in patients or care partners as identified by the different patterns. Future research should employ a dyadic approach to address the prevalence of characteristics and identify others to improve the mental health of both members of the dyad.

## 1. Introduction

Globally, chronic liver disease (CLD) and cirrhosis represent an underestimated major public health problem. An estimated 1.5 billion people have CLD—a number that is projected to increase over the next two decades [1]—and it accounts for 2 million deaths annually [2]. People with CLD experience significant disability and use of healthcare resources [2]. The most common causes of CLD include metabolic dysfunction-associated steatotic liver disease (MASLD), alcohol-related liver disease (ALD), chronic hepatitis B virus infection, and chronic hepatitis C virus infection [1].

In the United States (U.S.), 4.5 million people have CLD and an estimated 633,000 have cirrhosis [3]. Fewer than 11,500 people receive transplants and more than 52,000 die each year from the disease [3,4]. At the final stage of cirrhosis, also described as end-stage liver disease (ESLD), persons experience an average of 4.7 to 8.5 co-occurring symptoms, such as fatigue, difficulty sleeping, pain, uncertainty, and depression [5]. Unique complications of ESLD include ascites, hepatic encephalopathy, bacterial peritonitis, and variceal bleeds, which frequently lead to emergency department (ED) visits and hospitalization. Despite the complicated and uncertain disease trajectory and high symptom burden and healthcare resource use, patients diagnosed with ESLD rarely receive palliative care until late in the trajectory [6,7,8,9].

The burden regarding the complex care and needs of patients with ESLD is often left to their informal care partners, causing them to experience a high prevalence of anxiety, depression, uncertainty, poor physical and mental health, and diminished social function [10,11,12,13]. Care partners play a key role in helping patients with ESLD in performing both activities of daily living (ADLs) and instrumental activities of daily living (IADLs) [14]. Such activities may include bathing or showering, medication management and administration, medical appointment coordination, and transportation. The impact of palliative care has been examined in care partners for patients with cancer but rarely in care partners for patients with ESLD. In care partners for patients with cancer, early palliative care interventions showed an improvement in depression but mixed findings with regard to care partner burden [15].

ESLD affects the well-being and health of informal care partners and patients as individuals but also as an interdependent unit, often restricting their social lives [16,17]. Across illness contexts, health and well-being have been found to be interdependent (i.e., covarying) within the care dyad, and patients and care partners often influence one another’s outcomes [18]. Therefore, a dyadic approach to ESLD and other chronic diseases provides a more realistic picture of how patients and care partners influence the health of one another within the reciprocal nature of their relationship. Findings in limited cross-sectional dyadic research in ESLD showed that psychological distress was prevalent and interdependent among patients and their care partners and that patients experienced significantly worse mental and physical quality of life and higher levels of uncertainty than informal care partners, whereas care partners described the quality of their relationships as being significantly worse compared to patients [19,20]. In other chronic illness contexts, e.g., in cancer and stroke, longitudinal dyadic research has examined how health and well-being within care dyads change over time and its associations with outcomes [21,22]. This body of research has shown mental health to be particularly interdependent within dyads navigating an illness [21,22]. Although informal care partners are not a part of healthcare professionals’ clinical assessment and management of patients with ESLD, recognizing and understanding that mental health is interdependent within dyads is essential to advance clinical practice and improve disease management for patients with ESLD. It is important for healthcare professionals to understand that ESLD does not affect the patient alone but to be aware of the impact on the mental health of both members of the ESLD dyad and how it may change over time.

To our knowledge, there are no studies that have examined changes over time in mental health within ESLD patient–care partner dyads. Establishing mental health patterns is particularly important to identify the most vulnerable ESLD dyads where members of the dyad may be unable to support one another, are at risk for poorer outcomes, and therefore would benefit most from early, specifically tailored palliative care interventions. Thus, the aim of this study was to identify (1) distinct patterns of dyadic mental health (i.e., mental health of patients and care partners) over time in a sample of ESLD dyads and (2) associations with individual- and dyadic-level characteristics. We hypothesized that at least two types of patient–care partner dyads could be identified and differentiated based on patient-, care partner-, and dyad-level characteristics.

## 2. Materials and Methods

The analysis for this study was preregistered at the Open Science Framework (https://osf.io/jg2e7/overview?view_only=18af3a1ad81b4dc2a4c30d2e6254bc1a, accessed on 6 April 2024) and uses data from the Symptom Burden in End-Stage Liver Disease Patient–Caregiver Dyads study—a longitudinal, federally funded observational study of health in ESLD dyads [23].

### 2.1. Theoretical Framework

This work was guided by Lenz’s Theory of Unpleasant Symptoms [24]. This theory explains how one symptom can occur alone or how multiple symptoms can co-occur. The symptoms and characteristics included in this analysis were based on published research in patients with ESLD and their care partners, including symptoms (uncertainty), pathophysiological factors (liver disease severity), situational factors (patient and/or care partner predictor factors), and performance (health) within the context of the quality of the dyadic relationship.

### 2.2. Participants and Procedures

The sampling frame for this study was adult men and women with ESLD and their informal care partners. Care partners were an adult unpaid spouse or other non-blood relative (e.g., significant other, partner, close friend) or blood relative (e.g., parent, sibling, child, other relative) and identified by the patient as the primary care partner or support person. When patients and their care partners attended patients’ liver clinic appointments at an academic center and a veteran affairs medical center in the U.S. Pacific Northwest, patients were screened for study eligibility by one of 14 healthcare providers in the clinics. The two study sites are liver transplant centers and provide advanced care to patients with liver diseases. All 14 providers in the centers participated in the eligibility screening of patients.

Patients were eligible to participate in the study if they were 21 years of age or older and had a Model for End-Stage Liver Disease—Sodium (MELD-Na) score of 15 or greater. The MELD-Na is an established calculated formula used to determine short-term survival in patients with ESLD and to assign priority to liver transplant candidates (scores range from 6 to 40). Higher scores signify worse mortality, with scores from 10 to 19 predicting three-month survival to be 6 to 20% [25]. Patients were ineligible if they had a liver cancer diagnosis, had previously received liver transplantation, or were in active hepatitis C treatment. Care partners were eligible to participate in the study if they were 18 years of age or older. The patient–care partner dyad was excluded if either the patient or the care partner had a psychiatric illness resulting in disjointed thinking that prevented data collection or a major uncorrected hearing impairment. Both patients and care partners needed to be able to communicate by telephone with research team members if they had a depression score of 10 or greater.

After meeting with the healthcare provider, and if both members of the dyad were interested in learning more about the study and potentially participating, a study team member met with them immediately after the appointment. To facilitate recruitment, a designated team member was present during scheduled liver clinic appointments. The team member determined care partner study eligibility and, if both members were eligible, invited them to participate in the study. Patients and care partners were recruited consecutively when they attended patients’ liver clinic appointments. Prior to enrolling in the study, each member of the dyad provided informed written consent. If patients were unable to provide informed consent due to active hepatic encephalopathy, they and their care partners were contacted at a later clinic appointment.

After enrolling in the study, a survey packet that included a stamped envelope with a return address for completed surveys was provided to each member of the dyad separately. They had the option to complete the survey in the clinic or at home. If requested, surveys were administered in person or via telephone.

If the care partner of a study-eligible patient did not attend the clinic appointment with the patient, a research team member telephoned the care partner and described the study, determined eligibility, and invited them to participate in the study. If the care partner decided to participate, an informed consent form was included in the survey packet and mailed to them. Only dyads were enrolled in the study.

Data were collected at the time at which patients with ESLD and their care partners enrolled in the study, which was defined as baseline, and at 3, 6, 9, and 12 months. At baseline, each study participant completed a survey containing measures of uncertainty in illness, social support, relationship quality, religiosity, and mental and physical health. At the other four data collection points, the survey included the uncertainty in illness and mental and physical health measures. Prior to each data collection point, if patients were found to be encephalopathic based on care partner reports or patient comprehension regarding questions asked by research team members, data were not collected and surveys were mailed at a later date. Participants were compensated with USD 30 for each completed survey.

### 2.3. Measures

#### Demographics and Disease-Related Characteristics

Specific characteristics included in the statistical analysis were chosen based on our guiding theoretical framework and previous research. These were age, gender, comorbidities (measured by the Charlson Comorbidity Index) [26], type of care dyad (spousal versus non-spousal), and co-residence. Patient disease-related characteristics extracted from their medical records included MELD-Na scores and evidence of hepatic encephalopathy and ascites. The previously agreed upon characteristics and variables are listed below:

1. Type of care dyad (spousal versus non-spousal)
2. Patient age
3. Care partner age
4. Co-residence
5. Patient gender
6. Care partner gender
7. Stage of disease
  - Child–Turcotte–Pugh class
  - MELD 3.0
8. Patient comorbidities—using Charlson Comorbidity Index
9. Care partner comorbidities—using Charlson Comorbidity Index
10. Care partner strain
11. Care partner uncertainty in illness
12. Patient uncertainty in illness
13. Care partner physical health
14. Patient physical health
15. Care partner relationship quality
16. Patient relationship quality
17. Care partner social support
18. Patient social support
19. Care partner religiosity
20. Patient religiosity

### 2.4. Patient and Care Partner Variables

#### 2.4.1. Uncertainty in Illness

The 33-item Mishel Uncertainty in Illness Scale for Adults (MUIS-A) was used to measure uncertainty in illness in patients [27]. The 31-item Mishel Uncertainty in Illness Scale for Family Members (MUIS-FM) was used to measure care partners’ perceived uncertainty about the patient’s illness [28]. Both measures are scored on a 5-point Likert scale from strongly disagree = 1 to strongly agree = 5. The scores for each scale are totaled to a single score, with higher scores indicating higher levels of illness uncertainty. The MUIS-A and the MUIS-FM are highly reliable (sample α = 0.87 and α = 0.92, respectively) and valid and were chosen because they have been widely used with chronic illness samples [28]. In this study, sample α for the MUIS-A was 0.86 and that for the MUIS-FM was 0.88.

#### 2.4.2. Social Support

The 12-item Multidimensional Perceived Social Support Scale (MPSSS) was used to measure individual patients and care partners’ perceived levels of support from three sources: significant others, family members, and friends [29]. The scale uses a 7-point Likert scale from very strongly disagree = 1 to very strongly agree = 7. Higher scores indicate higher levels of perceived support. The scale is reliable (sample α = 0.90) and valid [30,31]. In this study, sample α was 0.93.

#### 2.4.3. Relationship Quality

The 15-item Mutuality Scale (MS) was used to measure the quality of the patient and care partner relationship [31]. The scale is scored on a 5-point Likert-type scale from not at all = 0 to a great deal = 4. The higher the score, the higher the quality of the relationship. Reliability (sample α = 0.90) and validity have been established for the scale [32,33]. In this study, reliability was 0.96 (sample α). The scale has been widely used in patients with chronic illnesses and care partners [34].

#### 2.4.4. Religiosity

Religiosity was measured on a 1-item, 5-point Likert-type scale from not religious = 1 to very religious = 5. Patients were asked to what extent they considered themselves to be religious. Higher scores indicated higher religiosity [35].

#### 2.4.5. Physical and Mental Health

The mental and physical components from the 36-item Short-Form Health Survey (SF-36) were used to measure patient and care partner mental and physical health. Scores were transformed to 0 to 100. The higher the score, the better the health. The SF-36 has well-established reliability (sample α = 0.85) and validity across populations [36,37,38]. In this study, sample α was 0.82.

### 2.5. Care Partner Variable

#### Care-Related Strain

The 18-item 5-point Multidimensional Caregiver Strain Index (MCSI) was used to measure care-related strain. It has six subscales: physical (3 items), social (4 items), financial (2 items), interpersonal strain (5 items), time constraints (2 items), and care receiver demands (2 items) [37]. The higher the score, the greater the strain. The MCSI has established reliability (sample α = 0.90) and validity with different types of care partners [39,40]. The sample α for this study was 0.90.

### 2.6. Statistical Analysis

Standard summary statistics were used to describe the study sample and to assess data integrity (e.g., ensure that there were no scale scoring errors). There was no further data manipulation (e.g., removing outliers) prior to running the hypothesized models. Although the MELD-Na score was used as an eligibility criterion in the original protocol, the updated MELD 3.0 score was used as a covariate in these analyses. Compared to the MELD-Na, the MELD 3.0 has been found to be a better predictor of mortality, particularly among biological females [41].

Formal statistical modeling proceeded in three steps using an integrative approach, as used previously by the study team [20]. First, multilevel modeling was used to generate empirical Bayes estimates of the dyadic mean mental health (i.e., average level of mental health within the dyad) and dyadic incongruence in mental health (i.e., difference in mental health between patient and care partner) for each wave of data collection to control for interdependencies in the dyadic data and autocorrelations over time. Summary scores for the mental component score (MCS) for both patient and partner at each wave were used, along with estimates of measurement error obtained using the known variance method [42]. Reliability estimates for the SF-36 MCS summary scores were taken from published estimates [43,44]. All multilevel modeling was conducted using the HLM 7 software [45]. Second, we used latent growth mixture modeling (as implemented in Mplus 8.7) to identify distinct patterns of change in dyadic mental health over time [46]. A latent categorical variable was estimated that represented distinct patterns of change in dyadic mental health as a function of the four parameters derived from the multilevel model described above (i.e., the intercept and slope of change in both the average level of mental health within the care dyad and the incongruence in mental health between members of the dyad over time). Standard criteria were then used to identify the number of patterns of change over time that best fit the observed data, including the proportion of cases in each pattern (should be no less than 5%), average classification probabilities (should be greater than 95%), model entropy calculated as a function of average classification probabilities (should be close to 1.0), the Lo–Mendell–Rubin Adjusted Likelihood Ratio Test (LMRALRT), and the parametric bootstrap likelihood ratio test (PBLRT) [47]. Models were fit up to a 6-class solution, and the best solution based on these criteria was chosen. After the best model solution was identified statistically, a full stop was embedded so that the core team of investigators could reach a consensus about the relevance and potential utility of the data-driven trajectories. Missing data were accounted for in Mplus by full information maximum likelihood estimation. Finally, to address the second part of the aim, we examined differences among the identified patterns of dyadic mental health over time with respect to prespecified demographic, clinical, and symptom variables using analysis of variance (for continuous variables) and chi-squared testing (for categorical variables) as appropriate.

## 3. Results

The sample (*N* = 186 dyads) included 81.4% White and 18.6% non-White individuals. The majority (40.3%) of the sample had completed some college, 18.3% had completed college, and 25.5% had graduated from high school. Most patients with ESLD and their care partners lived together (75.3%). Most care partners were female. Patients had a variety of liver disease etiologies and belonged to Child–Turcotte–Pugh classes B and C. Table 1 shows the sample demographics for the dyads included in this study. (See Supplementary Materials for flow diagram listing study patient–care partner dyad eligibility, recruitment, and attrition).

Latent growth mixture modeling revealed three distinct patterns of dyadic mental health over time (entropy = 0.91; LMRALRT, *p* = 0.01; PBLRT, *p* < 0.001); see Table 2.

Based on the characteristics of dyadic mental health over time, these three patterns were labeled as “disparate: patient better” (*n* = 47 [25.3%], average classification probability = 0.94), “shared mental health” (*n* = 76 [40.9%], average classification probability = 0.95), and “disparate: care partner better” (*n* = 63 [33.9%], average classification probability = 0.97).

### 3.1. Characteristics of Latent Patterns of Dyadic Mental Health

**Figure 1:**
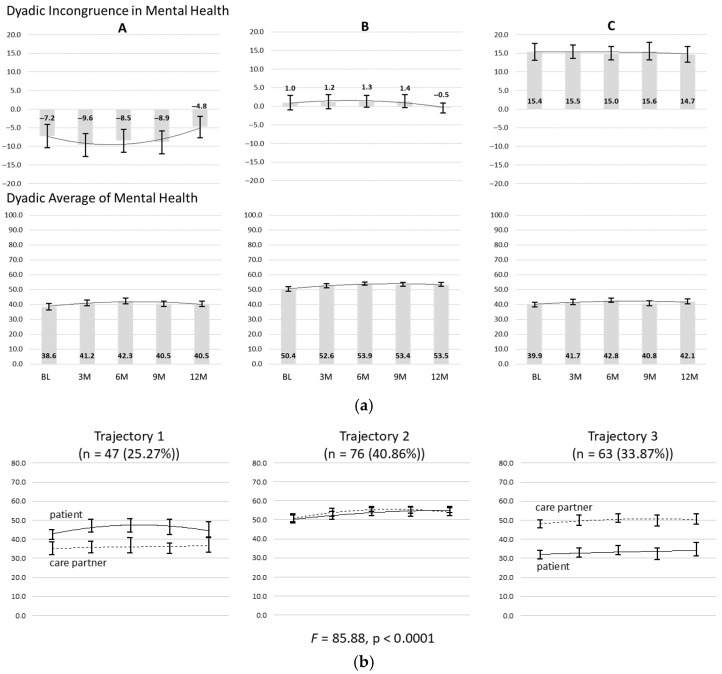
Distinct patterns of change in dyadic mental health over time. (**a**) presents the level of dyadic incongruence (i.e., the gap between the patient with ESLD and their care partner) and (**b**) presents the dyadic average (i.e., the mean between the patient with ESLD and their care partner) in mental health component summary scores on the SF-36.

Figure 1 presents both the incongruence in mental health between patients with ESLD and their care partners and the average mental health between members of the dyad for the three trajectory classes.

Column A presents data on the “disparate: patient better” pattern, where patients with ESLD (solid line) reported better mental health compared with their care partners (dashed line). Column B presents data on the “shared mental health” pattern, where patients with ESLD reported similar mental health compared with their care partners at moderate levels. Column C presents data on the “disparate: care partner better” pattern, where care partners reported better mental health compared with patients with ESLD.

There was a significant difference in dyadic incongruence in mental health (i.e., average difference in mental health between members of the care dyad) among the patterns at baseline (F(2, 183) = 85.88, *p* < 0.0001) that was sustained across 12 months. The “shared mental health” pattern (B) showed very little average difference in mental health between members of the dyad. The “disparate: patient better” pattern (A) indicated worse mental health for the care partner as compared to the patient with ESLD. The opposite and an even larger incongruence pattern occurred in the “disparate: care partner better” pattern (C), wherein patients with ESLD reported worse mental health across time as compared to their care partners.

There also was a significant difference in dyadic mental health (i.e., average level of mental health within the dyad) between the patterns at baseline (F(2, 183) = 59.56, *p* < 0.0001) that was sustained across 12 months. The disparate patterns (A and C) were very similar in terms of mean dyadic mental health. However, mean dyadic mental health was ~10 points greater in the “shared mental health” pattern (B) compared with the disparate patterns.

### 3.2. Associations with Sociodemographic and Clinical Characteristics

Table 1 shows all prespecified (a priori) study variables that were compared between the three trajectories. Table 3 shows the core characteristics of the three mental health trajectory classes. The “disparate: patient better” pattern, or the worse care partner mental health trajectory (class 1), was characterized by the smallest care partner comorbid burden, the highest care-related strain, higher illness uncertainty for the care partner and patient, the lowest care partner relationship quality, low patient relationship quality, low patient social support, and the lowest care partner religious importance. The “shared mental health” trajectory (class 2) was characterized by the lowest care-related strain, the lowest care partner and patient illness uncertainty, the best care partner and patient with ESLD relationship quality, the highest patient social support, and a high care partner religious importance rating. The “disparate: care partner better” trajectory (class 3) was characterized by higher care partner strain, higher care partner illness uncertainty, the highest patient illness uncertainty, low care partner relationship quality, the lowest patient relationship quality, the highest care partner comorbid burden, and a majority of non-spousal care partners.

## 4. Discussion

In this sample of ESLD dyads, three distinct patterns of dyadic mental health were found that were sustained over time. One pattern (B) displayed very little average difference in mental health between patients and care partners, whereas the other two patterns showed incongruence in mental health between them. In one pattern (A), care partners had worse mental health compared to patients. The opposite was evident in the other pattern (C). We observed that these three patterns could be identified and differentiated based on sociodemographic and clinical characteristics. These findings support our hypothesis and bring new insights into the longitudinal experience of mental health in ESLD dyads. To date, other studies conducted with a mental health focus have primarily been in chronic conditions, such as cancer [20,48], cardiac conditions [49,50], and stroke [51].

### 4.1. Characteristics of Latent Patterns of Dyadic Mental Health

Our study of dyadic mental health in ESLD dyads is one of few longitudinal studies focusing on dyadic patterns of mental health in the context of chronic illness. As in the study by Lee and Lyons [20], we found three distinct patterns of mental health in ESLD dyads. The authors studied a sample of 113 patients diagnosed with non-small-cell lung cancer and their care partners at 3, 6, 9, and 12 months [20]. They labeled their patterns congruent health (32.7%), disparate health (29.2%), and parallel health (38.1%). In both studies, mental health was measured with the SF-36. In contrast to the parallel health pattern, which showed an improvement in care partner mental health and greater similarities in mental health between patients and care partners over time, the other two patterns were similar to the ones that we found: the “disparate: patient better” pattern, in which patients reported better mental health than care partners, and “shared mental health”, in which little difference in mental health was reported between members of the care dyad and, on average, both reported moderate levels of mental health. Furthermore, in the parallel health pattern, care partners had worse mental health compared to patients. The opposite was found in our “disparate: care partner better” pattern, where patients had worse mental health compared to care partners. These differences in mental health, as illustrated by the different patterns and incongruence in the levels of mental health between patients and care partners in two of the patterns in our study and the study of Lee and Lyons, highlight the need to examine dyadic mental health in specific illness contexts to determine how both members of the dyad are experiencing mental health [20,52]. The significance of including specific types of dyadic patterns in intervention studies and clinical practice was also documented in the cross-sectional study by Chung et al. [53]. They examined depression, anxiety, health-promoting behaviors, and quality of life between patients with stroke and their care partners [53]. Among 147 dyads, the investigators identified four dyadic classes: collaborative-oriented (37.4%), patient-oriented (32.7%), incongruent (20.4%), and care partner-oriented (9.5%). The dyadic class was found to influence quality of life. It was higher in the collaborative and patient-oriented classes compared to the other two types.

In our study, in the “shared mental health” pattern, the mean dyadic mental health scores were 50.4–53.5, which are close to the normative U.S.-based value as defined by a mean score of 50 on the SF-36. This score is ~10 points greater compared to the average mental health in the disparate patterns. Translated into clinical practice, healthcare professionals should pay particular attention to care dyads in which one of the members has worse mental health, which may require additional screening and intervention to improve the mental health of the individual or both members of the dyad. This may be particularly true when the person with worse mental health is the care partner, as these dyads may be particularly at risk for poor outcomes when the care partner is also in need of intervention. In chronic and terminal illness, achieving similar mental health between members of the dyad may not be appropriate, possible, or feasible, particularly when shared mental health indicates that both members have poor as opposed to good mental health. Instead, the focus should be to obtain optimal and balanced mental health for each member and to pay particular attention when the mental health of one or both members is sub-optimal and in need of intervention.

### 4.2. Associations with Sociodemographic and Clinical Characteristics

Compared to the “patient better” and “care partner better” classes, the “shared mental health” class was characterized by the lowest care-related strain, the lowest care partner and patient illness uncertainty, the best care partner and patient with ESLD relationship quality, the highest patient social support, and higher care partner religious importance ratings. In this class, patients with ESLD and their care partners reported moderate levels of shared mental health, making this a desirable pattern as opposed to shared poor mental health. These findings are consistent with our theoretical framework of unpleasant symptoms, which shows that symptoms co-exist and that lower levels of uncertainty in illness and care-related strain would lead to better mental health in care dyads, with positive relationship quality often found to be a protective factor in patients and care partners [17]. Our findings did not reveal the underlying mechanisms for the characteristics associated with moderate levels of mental health in the “shared mental health” class. However, the broader field of dyadic health research consistently finds more positive relationships to be associated with better mental health outcomes in dyads, primarily through greater levels of collaboration, communication, and support within the relationship [54]. Thus, our findings align with dyadic health research examining mental health in other illness contexts, such as cancer and heart failure [20]. Associations with various sociodemographic and clinical characteristics have also been established in dyadic studies in chronic conditions [48,50]. Although the characteristics may be similar in studies but operationalized and measured differently, parallel findings may be noted with regard to our ESLD study. For example, the lowest care partner care-related strain and best relationship quality were found in the dyadic expert and collaborative classes compared to the novice and inconsistent classes in the heart failure study by Lee et al., who discovered three types of dyadic classes related to patient and care partner contributions to heart failure self-care [55].

Comparing the disparate classes, in the “patient better” (care partner worse) class, the patient reported low–moderate mental health, compared to the low mental health reported by patients in the “care partner better” class. The ratings of the care partners were opposite to those of patients with low mental health in the “patient better” class and moderate mental health in the “care partner better” class. Both classes were characterized by illness uncertainty. The “disparate: patient better” class, or the “care partner worse” class, was characterized by the smallest care partner comorbid burden, the highest care-related strain, the worst care partner relationship quality, and the lowest care partner religious importance. The “disparate: care partner better” class was characterized by the highest care partner comorbid burden and a majority of non-spousal care partners (e.g., adult children). The importance of examining the type of care partner–care recipient relationship was described by Ferraris et al. in their systematic review [18]. The authors concluded that understanding the dyadic interdependence among non-spousal caregiving dyads beyond spousal-focused research has important clinical implications for designing interventions to improve the well-being of both care partners and patients.

The differences in patients with ESLD’s MELD 3.0 scores and physical health in the “patient better” and “care partner better” classes were non-significant. However, care-related strain and the importance of religion were significant for care partners. The importance of religion as measured by a single item is an interesting finding. Among care partners for patients with dementia, higher religiosity and religious coping have been shown to lower the caregiver burden and reduce depressive symptoms [56,57]. In addition to care-related strain and religiosity, patient and care partner relationship quality and illness uncertainty were significantly associated with class membership. Future intervention studies could be designed to improve the mental health of both patients and care partners by decreasing illness uncertainty and care-related strain and improving relationship quality. Furthermore, future studies should explore the significance and importance of religiosity for ESLD care dyads using valid and reliable religiosity measures (e.g., the Centrality of Religiosity Scale (CRS)) [58].

Although dyadic interventions to address mental health have been studied in chronic conditions such as cancer [59,60], stroke [61,62], and Alzheimer’s disease [63], dyadic mental health research including intervention studies in ESLD is in its infancy. Two intervention studies without a specific focus on mental health have included patients with cirrhosis and their care partners. One study included an evaluation of a mindfulness-based stress reduction (MBSR) program [64]. The other study involved the testing of an uncertainty self-management telephone intervention [65]. This research shows promising new directions for a more dyadic approach to the support of ESLD care dyads and ways to reduce the prevalence of uncertainty and high levels of strain and burden experienced, both identified as potential characteristics for improving mental health in the current study.

Based on the differences among the three dyadic mental patterns in ESLD and their significant associations with sociodemographic and clinical characteristics found in this study, the evaluation of palliative care interventions to improve dyadic mental health is needed. ESLD is a life-limiting illness for which palliative care is developed. Palliative care is a patient- and family-centered approach that focuses on improving the quality of life of patients and their families by addressing their physical, psychosocial, or spiritual concerns or problems [66]. Thus, palliative care interventions focused on the care dyad may be particularly beneficial. The current study draws important attention to the impact of ESLD on both patients and their care partners and highlights distinct patterns of mental health within these dyads. Implications include greater attention in practice settings to both members of the dyad and particular attention to the needs of care partners, who themselves may require intervention to adequately support the patient. In addition to more dyadic approaches to clinical practice and the development and testing of interventions to reduce uncertainty and facilitate the dyad in finding ways to increase support and reduce care-related strain, future research is also needed to explore additional dyadic processes such as shared decision-making, collaborative illness management, and communication within the dyad, which may uncover additional mechanisms of change for optimal health outcomes. More broadly, larger dyadic studies that examine additional factors such as social determinants of health may further help to tailor interventions in effective ways. This study provides an important foundation for addressing the needs of both members of the ESLD dyad and highlights the need to tailor such interventions based on the risk levels of one or both members. It is this tailored and dyadic perspective that may lead to more optimal outcomes.

### 4.3. Clinical and Policy Implications

In clinical practice, to maintain or improve the mental health of each care partner and patient separately and interdependently, a focus of importance for healthcare professionals would be to conduct a general mental health or psychosocial assessment that includes, e.g., appearance, mood, intellectual ability, illness uncertainty, and religious importance. Dyads in all three mental health trajectory classes may benefit from a psychosocial assessment leading to specific recommendations or referrals to other healthcare professionals depending on patient and/or care partner needs. To address care partners’ low levels of mental health in the “patient better” trajectory (class 1), care-related strain, illness uncertainty, relationship quality, and religiosity may be of importance and in need of intervention. Referrals could be made to social work and mental health services. Social workers could help care partners to connect to chaplaincy and/or religious organizations in the community. To improve relationship quality for both care partners and patients, professional mental health counseling may be needed. Recommendations could include exploring care options for care partners to take a break (e.g., run errands, ask for help from family members and neighbors) from the challenges of caregiving. In the “care partner better” trajectory (class 3), the focus of healthcare professionals should be on patients’ low levels of mental health. Based on the current study’s findings, patient illness uncertainty is the highest and social support is the lowest. In addition to the mentioned recommendations and referrals, patients may benefit from receiving help from a social worker to connect to one-line and community-based support groups. For patients to manage illness uncertainty, research has shown that they require specific liver disease information provided by healthcare professionals. Patients wish to know about their disease progression and what to expect within the next 6 to 12 months [67]. To support or improve patients’ and care partners’ moderate levels of mental health in the “shared mental health” trajectory (class 2), healthcare professionals could explore and support what may be working for patients and care partners separately and as a dyad in relation to the characteristics associated with their mental health.

Palliative care referral should be considered by healthcare professionals and provided to patients with ESLD early in the disease course [68]. In collaboration with members of the liver team, palliative care interdisciplinary team members would have the expertise to address the problems—whether physical, psychosocial, or spiritual—of patients with ESLD and their care partners. The feasibility of a hepatology–specialty palliative care co-management program for outpatients with cirrhosis has been established [69].

The findings from our study show patterns of low and moderate levels of mental health in patients with ESLD and their care partners and sociodemographic and clinical characteristics associated with these patterns. These findings support the large body of research that demonstrates that the integration of mental health in health policies is essential. Such integration seeks to promote the well-being of individuals and families by promoting holistic support and care [70].

### 4.4. Strengths and Limitations

The strengths of the present study include the longitudinal design and large dyadic sample. It is one of few studies in ESLD dyads and the first to establish three distinct patterns of mental health. Limitations of this study include that the sample was mostly White and located in the U.S. Pacific Northwest, limiting the generalizability of these results. In addition, as in any observational study, there may be other unmeasured variables that have important associations with the observed latent patterns that we could not examine. Larger future studies may allow for the detection of additional trajectories of mental health over time among subgroups of ESLD dyads, including those with different characteristics related to social determinants of health. Our findings may be biased by the potentially unique characteristics of the patients and care partners who agreed to take part in the study. Finally, our measure of religiosity consisted of a single survey item, limiting the construct validity and reliability.

## 5. Conclusions

The findings from this longitudinal study bring new insights into and broaden our understanding of the longitudinal experience of mental health patterns in ESLD dyads. Three distinct patterns revealed low and moderate levels of mental health in patients and care partners that were sustained over time. These sustained levels of mental health need intervention in clinical practice. Healthcare professionals should assess mental health in each member of the dyad individually and as a unit and make referrals as needed, including to palliative care. Future research should employ a dyadic approach and explore dyadic processes (e.g., communication) within the dyad, with the goal of developing interventions to improve mental health in ESLD dyads.

## Figures and Tables

**Table 1:** Sample demographic characteristics for 186 dyads.

Characteristic	*N* = 186	MH Traj 1 *n* = 47 (25.3%)	MH Traj 2 *n* = 76 (40.9%)	MH Traj 3 *n* = 63 (33.9%)	F/χ^2^, *p*-Value
Spousal vs. not	109 (58.6%)	32 (68.1%)	50 (65.8%)	27 (42.9%)	9.80, 0.007
Patient age (years)	56.4 (±11.0)	57.4 ± 11.9	57.2 ± 10.9	54.7 ± 10.5	1.14, 0.321
Care partner age (years)	56.7 (±13.2)	53.2 ± 15.1	57.7 ± 10.7	58.3 ± 13.9	2.38, 0.095
Cohabitating	140 (75.3%)	38 (80.6%)	58 (76.3%)	44 (69.8%)	1.83, 0.401
Female patient	64 (34.4%)	13 (27.7%)	26 (34.2%)	25 (39.7%)	1.73, 0.422
Female care partner	140 (75.3%)	35 (74.5%)	55 (72.4%)	50 (79.4%)	0.93, 0.629
Child–Turcotte–Pugh Class					
Class A	6 (3.2%)	1 (2.1%)	3 (4.0%)	2 (3.2%)	
Class B	86 (46.5%)	23 (48.9%)	32 (42.7%)	31 (49.2%)	0.95, 0.917
Class C	93 (50.3%)	23 (48.9%)	40 (53.3%)	30 (47.6%)	
MELD 3.0	19.1 ± 5.0	18.9 ± 5.2	19.2 ± 5.1	19.1 ± 4.6	0.05, 0.956
Patient Charlson Index	3.7 ± 1.9	3.7 ± 1.8	3.4 ± 1.8	4.1 ± 2.0	2.85, 0.061
Care partner Charlson Index	1.0 ± 1.2	0.6 ± 0.8	1.1 ± 1.3	1.2 ± 1.2	4.09, 0.018
Care-related strain (MCSI total)	17.3 ± 11.3	24.3 ± 12.5	12.9 ± 8.7	17.3 ± 10.8	17.13, <0.001
Care partner uncertainty (MUIS total)	82.7 ± 15.8	86.4 ± 14.8	78.1 ± 15.7	85.4 ± 15.6	5.78, 0.004
Patient uncertainty (MUIS total)	89.2 ± 16.4	90.0 ± 12.2	80.4 ± 15.3	99.3 ± 14.3	30.56, <0.001
Care partner physical health (SF-12 PCS)	48.3 ± 10.5	49.2 ± 10.9	48.2 ± 10.4	47.8 ± 10.6	0.23, 0.797
Patient physical health (SF-12 PCS)	33.1 ± 9.9	33.0 ± 10.1	34.7 ± 10.6	31.3 ± 8.6	1.99, 0.140
Care partner relationship quality (total score)	3.0 ± 0.8	2.7 ± 0.9	3.3 ± 0.6	3.0 ± 0.9	7.29, 0.001
Patient relationship quality (total score)	3.2 ± 0.8	3.2 ± 0.7	3.5 ± 0.6	3.0 ± 0.8	7.50, 0.001
Care partner social support (MPSSS)	66.6 ± 14.6	63.8 ± 14.1	66.2 ± 15.1	68.1 ± 14.3	1.28, 0.282
Patient social support (MPSSS)	67.5 ± 14.6	66.6 ± 12.5	72.2 ± 11.6	62.4 ± 17.4	8.42, 0.003
Care partner importance of religion	2.3 ± 1.5	1.8 ± 1.4	2.5 ± 1.6	2.4 ± 1.4	3.77, 0.025
Patient importance of religion	2.2 ± 1.5	2.0 ± 1.6	2.2 ± 1.6	2.4 ± 1.4	0.77, 0.463

Abbreviations. MH Traj, Mental Health Trajectory; MELD 3.0, Model for End-Stage Liver Disease; MCSI, Multidimensional Caregiver Strain Index; MUIS, Mishel Uncertainty in Illness Scale; SF-12 PCS, Short-Form Physical Component Score; MPSSS, Multidimensional Perceived Social Support Scale.

**Table 2:** Latent growth mixture model 2–6-class solutions.

	2 Classes	3 Classes	4 Classes	5 Classes	6 Classes
Class size	89, 97	47, 76, 63	16, 60, 45, 65	29, 15, 46, 50, 46	11, 17, 17, 47, 50, 44
Entropy	0.877	0.907	0.939	0.925	0.941
Probabilities	0.963, 0.965	0.940, 0.954, 0.975	0.994, 0.986, 0.934, 0.964	0.919, 0.972, 0.929, 0.953, 0.972	0.997, 0.976, 0.989, 0.927, 0.954, 0.970
LMRALRT	*p* = 0.182	*p* = 0.012	*p* = 0.063	*p* = 0.434	*p* = 0.301
PBLRT	*p* < 0.001	*p* < 0.001	*p* < 0.001	*p* < 0.001	*p* < 0.001

Abbreviations. LMRALRT, Lo–Mendell–Rubin Adjusted Likelihood Ratio Test; PBLRT, parametric bootstrap likelihood ratio test.

**Table 3:** Core characteristic profiles of the three trajectory classes.

MH Traj Class 1	MH Traj Class 2	MH Traj Class 3
Majority Spousal CP	Majority Spousal CP	Majority of Non-Spousal CP
Lowest Comorbid Burden (CP)	Higher Comorbid Burden (CP)	Highest Comorbid Burden (CP)
Highest Care-Related Strain (CP)	Lowest Care-Related Strain (CP)	Higher Care-Related Strain (CP)
Higher Illness Uncertainty (CP)	Lowest Illness Uncertainty (CP)	Higher Illness Uncertainty (CP)
Higher Illness Uncertainty (PT)	Lowest Illness Uncertainty (PT)	Highest Illness Uncertainty (PT)
Lowest Relationship Quality (CP)	Highest Relationship Quality (CP)	Lower Relationship Quality (CP)
Lower Relationship Quality (PT)	Highest Relationship Quality (PT)	Lowest Relationship Quality (PT)
Lower Social Support (PT)	Highest Social Support (PT)	Lowest Social Support (PT)
Lowest Religious Importance (CP)	Higher Religious Importance (CP)	Higher Religious Importance (CP)

Abbreviations. MH Traj, Mental Health Trajectory; CP, care partner; PT, patient.

## Data Availability

The data presented in this study are available on request from the corresponding author due to legal reasons.

## Supplementary Materials

The following supporting information can be downloaded at https://www.mdpi.com/article/10.3390/healthcare14050645/s1, Figure S1: Study patient–care partner dyad eligibility, recruitment, and attrition.

## Abbreviations

The following abbreviations are used in this manuscript:

CLD	Chronic liver disease
MASLD	Metabolic dysfunction-associated steatotic liver disease
ALD	Alcohol-related liver disease
ESLD	End-stage liver disease
ED	Emergency department
ADLs	Activities of daily living
IADLs	Instrumental activities of daily living
MELD-Na	Model for End-Stage Liver Disease—Sodium
MUIS-A	Mishel Uncertainty in Illness Scale for Adults
MUIS-FM	Mishel Uncertainty in Illness Scale for Family Members
MPSSS	Multidimensional Perceived Social Support Scale
MS	Mutuality Scale
SF-36	Short-Form Health Survey
MCS	Mental Component Score
MCSI	Multidimensional Caregiver Strain Index
HLM	Hierarchical linear modeling
LMRALRT	Lo–Mendell–Rubin adjusted likelihood ratio test
PBLRT	Parametric bootstrap likelihood ratio test
MH	Mental health
PCS	Physical Component Score
HRQOL	Health-related quality of life
