# Supplementary material for: Distinct Patterns of Dyadic Mental Health in Patients with End-Stage Liver Disease and Their Care Partners
Source: Healthcare (Basel). 2026 Mar 4;14(5):645. doi: 10.3390/healthcare14050645 (PMC12984372; doi:10.3390/healthcare14050645)
Supplement: Supplementary file 1 [file healthcare-14-00645-s001.zip › healthcare-4123638-supplementary.pdf]

Figure S1. Study Patient-Care Partner Dyad Eligibility, Recruitment, and Attrition

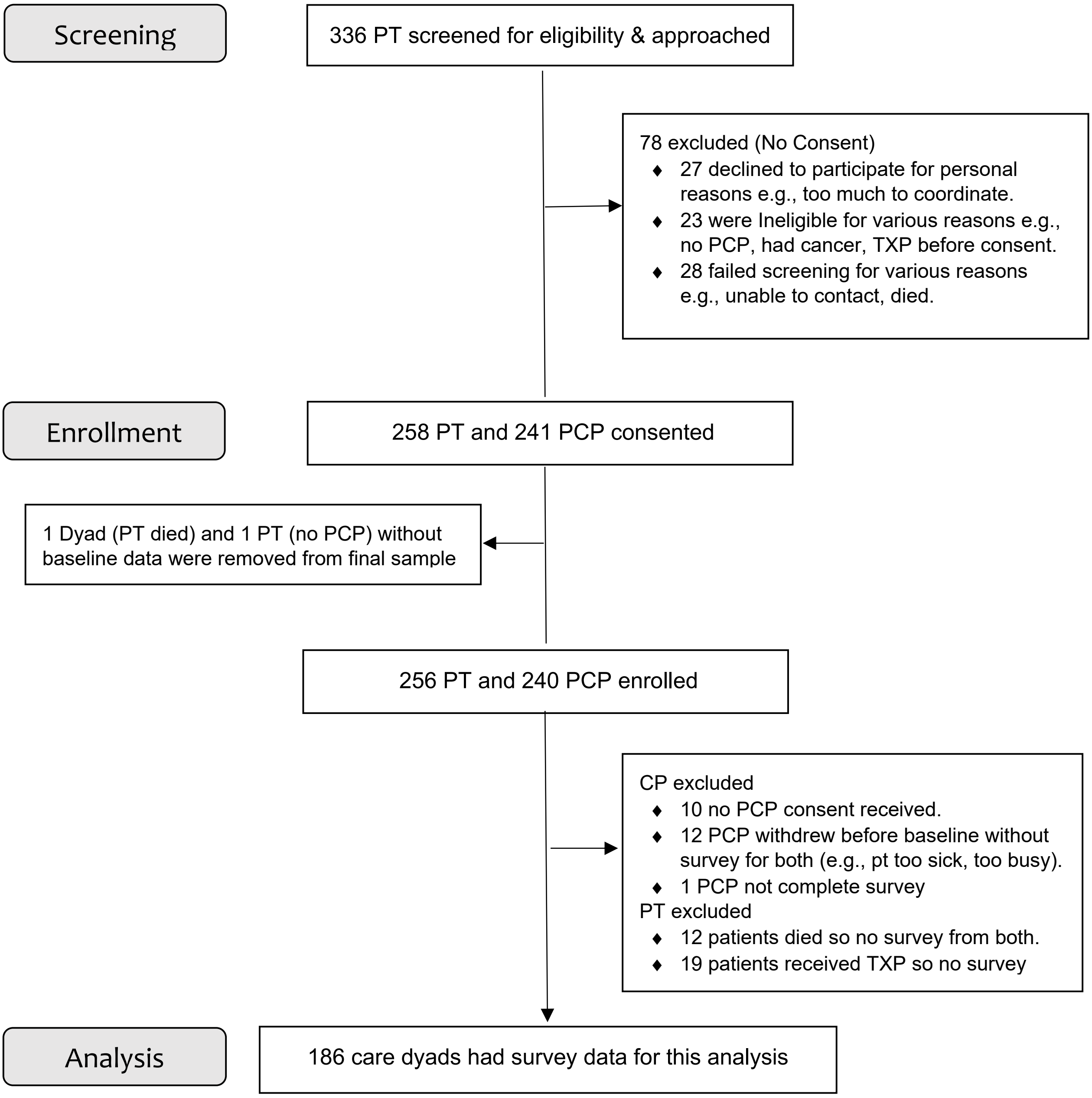

Note. PCG, Primary Care partner; PT: Patient; TXP, Transplantation.

Data presented in this article are patient and care partner reported. Data were collected from each member separately.
